# Supplementary material for: Assessing protected areas as climate refugia for threatened plant species in Britain
Source: PLoS One. 2026 Jan 23;21(1):e0332485. doi: 10.1371/journal.pone.0332485 (PMC12829861; doi:10.1371/journal.pone.0332485)
Supplement: S1 Table — This includes their scientific and common names, species descriptions [1], broad habitat types [2], and corresponding UK CEH land cover classes and identifiers [3]. (PDF) [file pone.0332485.s001.pdf]

Table S1. Summary information of the species included in this study. This includes their scientific and common names, species descriptions [1], broad habitat types [2], and corresponding UK CEH land cover classes and identifiers [3].

Scientific name	Common name	Description	Broad Habitat	UK CEH Land Cover Class	Land Cover Identifier
Adonis annua L.	Pheasant's-eye	An arable plant whose population is small and largely restricted to field edges.	4	Arable	3
Turritis glabra L.	Tower Mustard	A biennial herb found in periodically disturbed marginal habitats. Populations are small and isolated from each other.	8	Acid grassland	7
Carex ericetorum Pollich	Rare Spring-sedge	A perennial sedge of short grassland on infertile soils. A poor competitor and is declining in distribution.	7	Heather	6
Cerastium alpinum L.	Alpine Mouse-ear	A perennial herb of montane habitats. Evidence that its distribution has declined.	15, 16	Acid grassland, Heather grassland, Inland rock	7, 10, 12
Dryas octopetala L.	Mountain Avens	A dwarf creeping shrub. Populations are very small but distribution appears stable.	7, 16	Inland rock, Calcareous grassland	12, 6
Galeopsis angustifolia Ehrh. Ex Hoffm.	Red Hemp-nettle	An annual herb that has declined in distribution.	4, 16	Arable, Inland rock	3, 12
Juniperus communis L.	Common Juniper	A dioecious evergreen conifer of varied habitats. Declining due to grazing, burning, disease, and habitat loss.	7, 10, 15, 16	Calcareous grassland, Heather grassland, Acid grassland, Inland rock	6, 10, 7, 12
Mertensia maritima (L.) Gray	Oysterplant	A perennial herb of beaches and shingle, with recent northward expansion and southern range contraction.	19	Supralittoral sediment	16
Bistorta vivipara (L.) Delarbre	Alpine Bistort	A short, tufted perennial herb with a stable core range but possible decline at the edges of its distribution.	7, 15	Calcareous grassland, Acid grassland, Heather grassland	6, 7, 10
Ranunculus tripartitus DC.	Three-lobed Crowfoot	An annual herb of shallow water bodies, experiencing a gradual decline due to habitat loss.	13	Freshwater	14
Silene conica L.	Sand Catchfly	An annual herb that has declined throughout Britain. Remaining populations are often small and vulnerable.	8, 19	Acid grassland, Supralittoral sediment	7, 16
Spiranthes romanzoffiana Cham.	Irish Lady's-tresses	A tuberous herb with a wide yet scattered distribution.	11	Fen	8

References:

1. Stroh PA, Walker KJ, Humphrey TA, Pescott OL. Plant atlas 2020: mapping changes in the distribution of the British and Irish flora. 2023.
2. Hill MO, Preston CD, Roy DB. PLANTATT: attributes of British and Irish plants. 2004.
3. Marston CG, O'Neil AW, Morton RD. LCM2021–the UK land cover map 2021. Earth Syst Monit. 2023.
