# Supplementary material for: Assessing protected areas as climate refugia for threatened plant species in Britain
Source: PLoS One. 2026 Jan 23;21(1):e0332485. doi: 10.1371/journal.pone.0332485 (PMC12829861; doi:10.1371/journal.pone.0332485)
Supplement: S1 Text — (PDF) [file pone.0332485.s002.pdf]

**S1 Text. Refinement of species distribution based on habitat suitability**

We used the 25 m rasterized version of the UKCEH Land Cover Map for 2021 to refine species distribution [1]. This is a land cover classification that divides satellite images into 21 broad habitats [2]. We identified the preferred habitat types of the focal plants using Plantatt [3], with the corresponding land cover classes for each species listed in Table S1.

For each species, we identified species-specific suitable habitats and combined land cover maps indicating preferences with the projections of climate suitability to generate current and projected future habitat and climate suitability at 25 m resolution. To address the resolution differences between the 10 km SDM's and the 25 m habitat data we disaggregated the SDM estimates with no downscaling so that it matched the finer resolution.

The UKCEH Land Cover Map does not overlap with the period of the SDM. This is because the land cover maps were used after the SDM process as a spatial filter, to ensure that both current and future climate suitability are limited to habitat types that are currently present, based on the most recent land-cover information.

**References:**

1. Marston CG, O'Neil AW, Morton RD. LCM2021–the UK land cover map 2021. Earth Syst Monit [Internet]. 2023; Available from: https://essd.copernicus.org/articles/15/4631/2023/
2. Price C, Elsner P. Uncertainty of Historic GLAD Forest Data in Temperate Climates and Implications for Forest Change Modelling. ISPRS International Journal of Geo-Information. 2022;11: 177. doi:10.3390/ijgi11030177
3. Hill MO, Preston CD, Roy DB. PLANTATT-attributes of British and Irish plants: status, size, life history, geography and habitats. Centre for Ecology & Hydrology; 2004.
