# Supplementary material for: Assessing protected areas as climate refugia for threatened plant species in Britain
Source: PLoS One. 2026 Jan 23;21(1):e0332485. doi: 10.1371/journal.pone.0332485 (PMC12829861; doi:10.1371/journal.pone.0332485)
Supplement: S1 Fig — Green represents PAs where the species have in situ refugia, blue denotes ex situ refugia, and red indicates areas where the species is at risk. PAs that do not fall into these categories are not shown. (PDF) [file pone.0332485.s003.pdf]

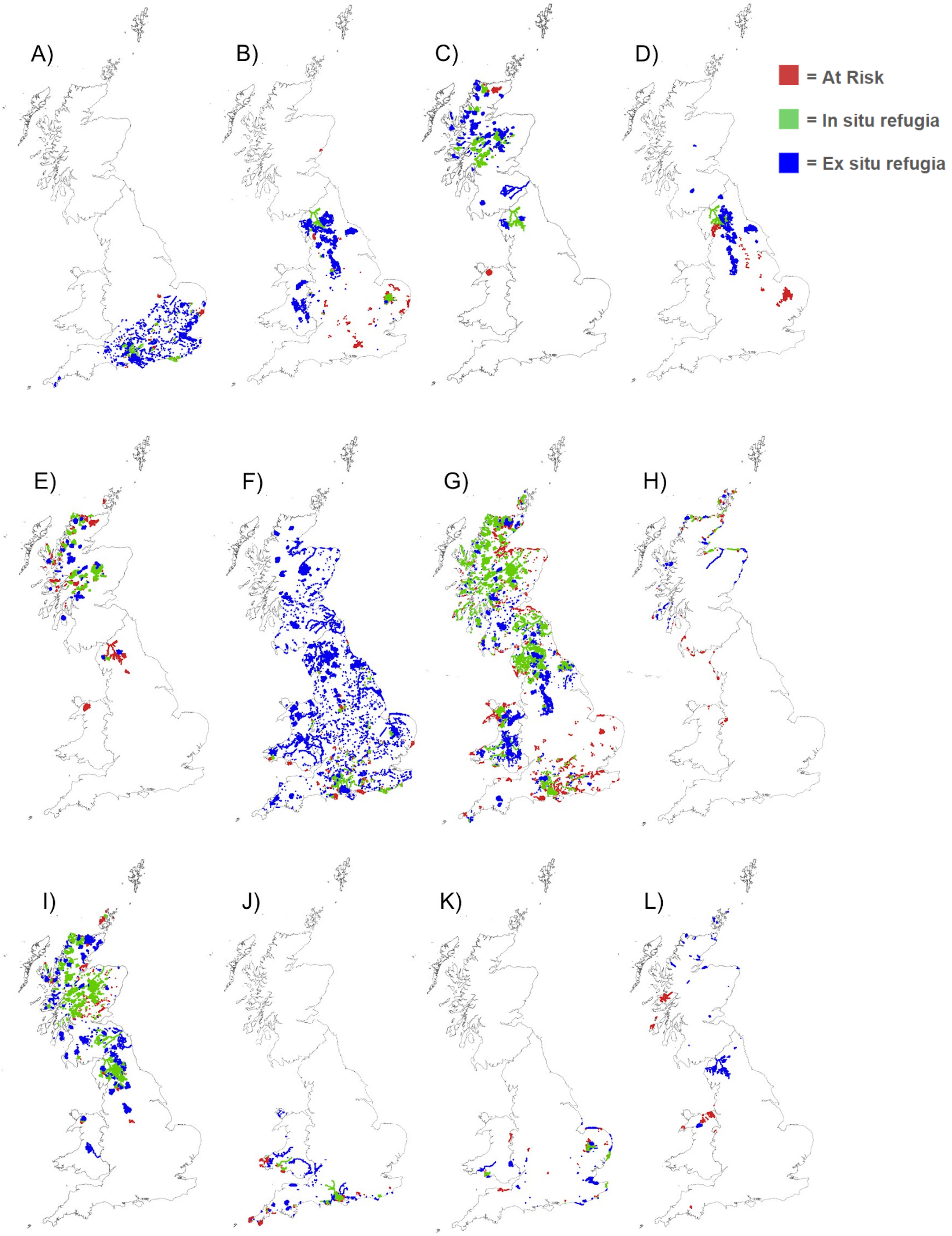

Fig S1. Maps illustrating the distribution of in situ refugia, ex situ refugia and areas at risk in each NNR and SSSI across England, Scotland and Wales. Green represents PAs

where the species have in situ refugia, blue denotes ex situ refugia, and red indicates areas where the species is at risk. PAs that do not fall into these categories are not shown.

- **A)** *Adonis annua* L.
- **B)** *Turritis glabra* L.
- **C)** *Carex ericetorum* Pollich
- **D)** *Cerastium alpinum* L.
- **E)** *Dryas octopetala* L.
- **F)** *Galeopsis angustifolia* Ehrh. Ex Hoffm.
- **G)** *Juniperus communis* L.
- **H)** *Mertensia maritima* (L.) Gray
- **I)** *Bistorta vivipara* (L.) Delarbre
- **J)** *Ranunculus tripartitus* DC.
- **K)** *Silene conica* L.
- **L)** *Spiranthes romanzoffiana* Cham.
