# Supplementary material for: Assessing protected areas as climate refugia for threatened plant species in Britain
Source: PLoS One. 2026 Jan 23;21(1):e0332485. doi: 10.1371/journal.pone.0332485 (PMC12829861; doi:10.1371/journal.pone.0332485)
Supplement: S2 Fig — The covariates included are latitude (A and B) and longitude (C and D). Figures on the left show the relationships with the number of species with in situ refugia, while those on the right depict species with ex situ refugia in each PA. Lines of best fit derived from a Bayesian generalised linear mixed model are represented with 95% credible intervals (green for in situ refugia and blue for ex situ refugia). All graphs show a significant positive correlation, except for the one between latitude and the number of species with in situ refugia, which shows no correlation. (PDF) [file pone.0332485.s004.pdf]

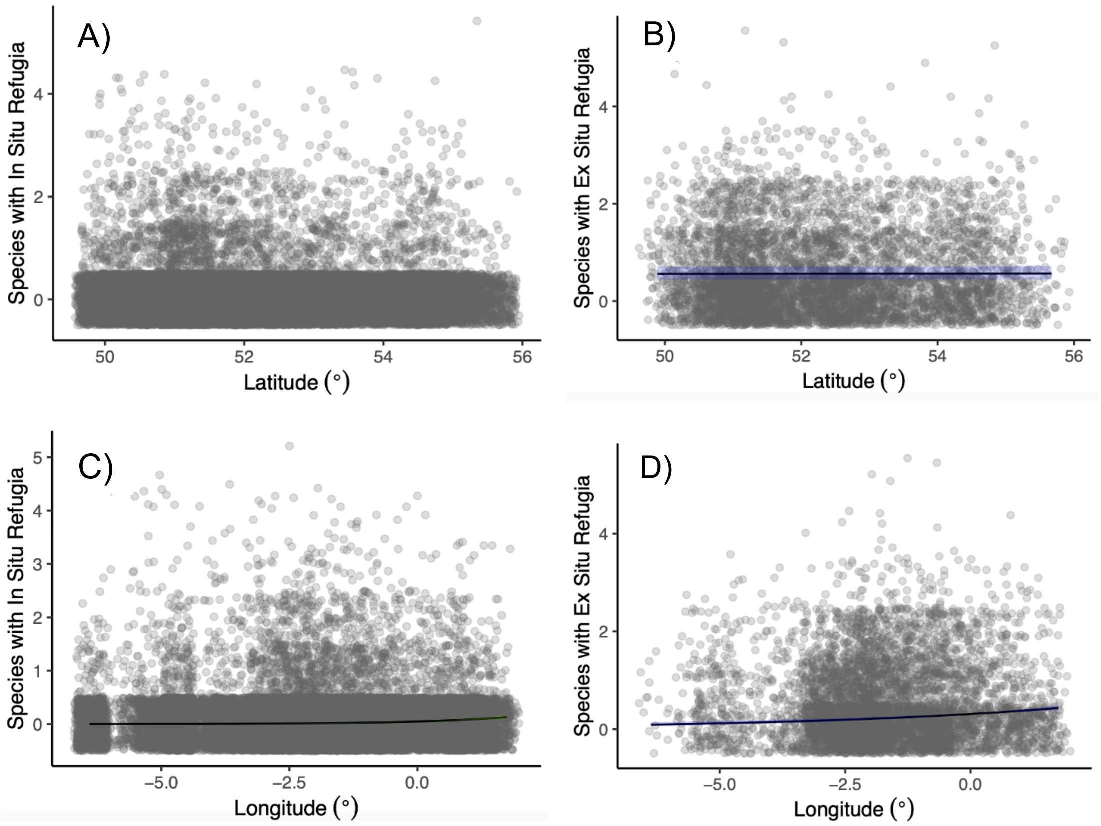

Fig S2. Effect plots illustrating the relationship between PA covariates (latitude (a & b) and longitude (c & d)) and the number of species finding refugia within the PA. Figures on the left show the relationships with the number of species with in situ refugia, while those on the right depict species with ex situ refugia in each PA. Lines of best fit derived from a Bayesian generalised linear mixed model are represented with 95% credible intervals (green for in situ refugia and blue for ex situ refugia). All graphs show a significant positive correlation, except for the one between latitude and the number of species with in situ refugia, which shows no correlation.
