# Supplementary material for: Assessing protected areas as climate refugia for threatened plant species in Britain
Source: PLoS One. 2026 Jan 23;21(1):e0332485. doi: 10.1371/journal.pone.0332485 (PMC12829861; doi:10.1371/journal.pone.0332485)
Supplement: S3 Fig — Graph (A) illustrates species with in situ refugia, while graph (B) depicts those with ex situ refugia. Null data points were omitted. Neither graph identified a correlation between the number of species with refugia and the number of habitat types. (PDF) [file pone.0332485.s005.pdf]

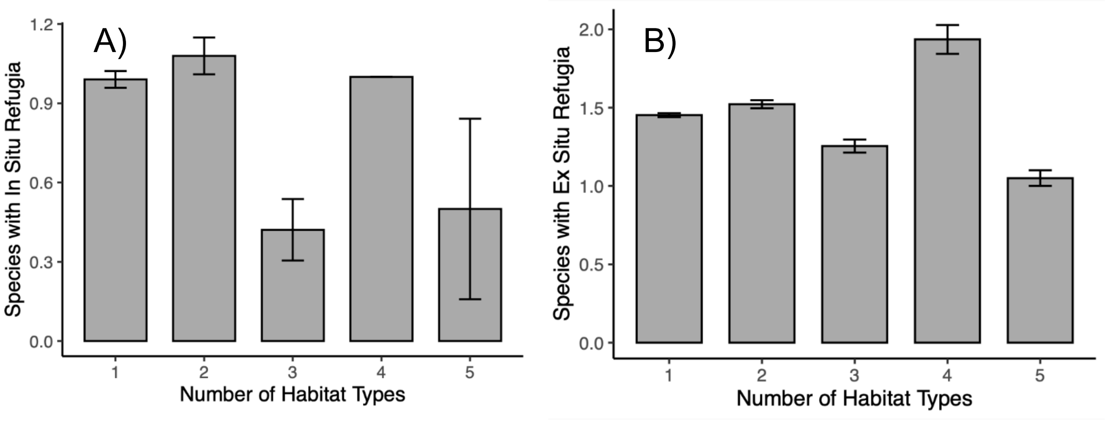

Fig S3. Bar graphs illustrating the number of habitat types within each PA alongside the number of species with refugia. Graph (a) illustrates species with in situ refugia, while graph (b) depicts those with ex situ refugia. Null data points were omitted. Neither graph identified a correlation between the number of species with refugia and the number of habitat types.
