# Supplementary material for: Assessing protected areas as climate refugia for threatened plant species in Britain
Source: PLoS One. 2026 Jan 23;21(1):e0332485. doi: 10.1371/journal.pone.0332485 (PMC12829861; doi:10.1371/journal.pone.0332485)
Supplement: S2 Text — (PDF) [file pone.0332485.s006.pdf]

S2 Text. Plantlife nature reserves case study.

We also looked at Plantlife Nature Reserves for a separate case study, using data provided by Plantlife to inform future conservation work for their focal species. Plantlife have been carrying out translocations across Britain, and it is crucial to consider future climate change when carrying these out. This case study also demonstrates how this analysis can be applied on a more practical scale.

We repeated the previous methods using Plantlife reserves instead of SSSIs and NNRs. This enabled us to identify Plantlife reserves that have current and future suitability for present species, as well as reserves with potential climate space for species currently absent, suggesting viable introduction sites. Additionally, we identified species that are currently in a reserve but are not projected to have both current and future suitability.

Information from the case study on the Plantlife Nature Reserves including species suitability, current presence, potential translocation opportunities and species considered at risk for each reserve are also available in the supplementary information (S2-S3 Tables).
