# Supplementary material for: Assessing protected areas as climate refugia for threatened plant species in Britain
Source: PLoS One. 2026 Jan 23;21(1):e0332485. doi: 10.1371/journal.pone.0332485 (PMC12829861; doi:10.1371/journal.pone.0332485)
Supplement: S2 Table — The “Suitable_score” column is the probability of the reserve being suitable now and in the future. The “Suitable” column indicates if this value is above the suitability threshold. The “Presence” column indicates if this species is already there. “Introduction” indicates if the reserves are suitable for the species but the species is not currently present, indicating opportunities for species introduction. “At Risk” indicates species that are present but do not have both current and future suitability. (PDF) [file pone.0332485.s007.pdf]

Table S2. Presence and suitability of focal species for Plantlife's reserves, arranged by reserve. The "Suitable_score" column is the probability of the reserve being suitable now and in the future. The "Suitable" column indicates if this value is above the suitability threshold. The "Presence" column indicates if this species is already there. "Introduction" indicates if the reserves are suitable for the species but the species is not currently present, indicating opportunities for species introduction. "At Risk" indicates species that are present but do not have both current and future suitability.

Reserve	Species	Suitable	Suitable score	Presence	Introduction	At Risk
Augill Pasture	*J. communis*	True	0.115303	False	True	False
Augill Pasture	*C. ericetorum*	True	0.039578	False	True	False
Augill Pasture	*B. vivipara*	True	0.004584	True	False	False
Cae Blaen-dyffryn	*J. communis*	True	0.004561	False	True	False
Caeau Tan y Bwlch	*J. communis*	False	0	True	False	True
Deep Dale	*J. communis*	True	0.007052	False	True	False
Deep Dale	*C. ericetorum*	True	0.001057	False	True	False
Deep Dale	*G. angustifolia*	False	0	True	False	True
Greena Moor	*G. angustifolia*	True	9x10^{-6}	False	True	False
Ranscombe Farm Reserve	*A. annua*	True	0.004922	False	True	False
Ranscombe Farm Reserve	*G. angustifolia*	True	3x10^{-6}	False	True	False
Ranscombe Farm Reserve	*J. communis*	False	0.002328	True	False	True
Ranscombe Farm Reserve	*J. communis*	False	0.001542	True	False	True
Side Farm Meadows	*J. communis*	True	0.004224	False	True	False
Skylark Meadows	*A. annua*	True	0.003844	False	True	False
Thompson Meadow	*G. angustifolia*	True	4.8x$^{-0.5}$	False	True	False
Thompson Meadow	*C. ericetorum*	False	0	True	False	True
Upton Ham	*J. communis*	False	0	True	False	True
Winskill Stones	*A. annua*	True	0.000330	False	True	False
Winskill Stones	*J. communis*	True	0.056874	True	False	False
Winskill Stones	*B. vivipara*	False	0.000212	True	False	True
