# Supplementary material for: Assessing protected areas as climate refugia for threatened plant species in Britain
Source: PLoS One. 2026 Jan 23;21(1):e0332485. doi: 10.1371/journal.pone.0332485 (PMC12829861; doi:10.1371/journal.pone.0332485)
Supplement: S3 Table — The “Suitable_score” column is the probability of the reserve being suitable now and in the future. The “Suitable” column indicates if this value is above the suitability threshold. The “Presence” column indicates if this species is already there. “Introduction” indicates if the reserves are suitable for the species but the species is not currently present, indicating opportunities for species introduction. “At Risk” indicates species that are present but do not have both current and future suitability. (PDF) [file pone.0332485.s008.pdf]

Table S3. Presence and suitability of focal species for Plantlife's reserves, arranged by reserve. The "Suitable_score" column is the probability of the reserve being suitable now and in the future. The "Suitable" column indicates if this value is above the suitability threshold. The "Presence" column indicates if this species is already there. "Introduction" indicates if the reserves are suitable for the species but the species is not currently present, indicating opportunities for species introduction. "At Risk" indicates species that are present but do not have both current and future suitability.

Species	Reserve	Suitable	Suitable score	Presence	Introduction	At Risk
A. annua	Ranscombe Farm Reserve	True	0.004922	False	True	False
A. annua	Skylark Meadows	True	0.003844	False	True	False
A. annua	Winskill Stones	True	0.00033	False	True	False
C. ericetorum	Augill Pasture	True	0.039578	False	True	False
C. ericetorum	Deep Dale	True	0.001057	False	True	False
C. ericetorum	Thompson Meadow	False	0	True	False	True
G. angustifolia	Thompson Meadow	True	$4.8x10^{-5}$	False	True	False
G. angustifolia	Greena Moor	True	$9x10^{-6}$	False	True	False
G. angustifolia	Ranscombe Farm Reserve	True	$3x10^{-6}$	False	True	False
G. angustifolia	Deep Dale	False	0	True	False	True
J. communis	Augill Pasture	True	0.115303	False	True	False
J. communis	Deep Dale	True	0.007052	False	True	False
J. communis	Cae Blaen-dyffryn	True	0.004561	False	True	False
J. communis	Side Farm Meadows	True	0.004224	False	True	False
J. communis	Winskill Stones	True	0.056874	True	False	False
J. communis	Ranscombe Farm Reserve	False	0.002328	True	False	True
J. communis	Ranscombe Farm Reserve	False	0.001542	True	False	True
J. communis	Upton Ham	False	0	True	False	True
J. communis	Caeau Tan y Bwich	False	0	True	False	True
B. vivipara	Augill Pasture	True	0.004584	True	False	False
B. vivipara	Winskill Stones	False	0.000212	True	False	True
